# Comparative assessment of favipiravir and remdesivir against human coronavirus NL63 in molecular docking and cell culture models

**DOI:** 10.1038/s41598-021-02972-y

**Published:** 2021-12-06

**Authors:** Yining Wang, Pengfei Li, Sajjan Rajpoot, Uzma Saqib, Peifa Yu, Yunlong Li, Yang Li, Zhongren Ma, Mirza S. Baig, Qiuwei Pan

**Affiliations:** 1 Department of Gastroenterology and Hepatology, Erasmus MC-University Medical Center, Room Na-1005, Wytemaweg 80, 3015 CN Rotterdam, The Netherlands; 2 Department of Biosciences and Biomedical Engineering (BSBE), Indian Institute of Technology Indore (IITI), Simrol, Indore, 453552 MP India; 3 Department of Chemistry, Indian Institute of Technology Indore (IITI), Simrol, Indore, 453552 MP India; 4 Biomedical Research Center, Northwest Minzu University, Lanzhou, China

**Keywords:** Drug discovery, Microbiology

## Abstract

Human coronavirus NL63 (HCoV-NL63) mainly affects young children and immunocompromised patients, causing morbidity and mortality in a subset of patients. Since no specific treatment is available, this study aims to explore the anti-SARS-CoV-2 agents including favipiravir and remdesivir for treating HCoV-NL63 infection. We first successfully modelled the 3D structure of HCoV-NL63 RNA-dependent RNA polymerase (RdRp) based on the experimentally solved SARS-CoV-2 RdRp structure. Molecular docking indicated that favipiravir has similar binding affinities to SARS-CoV-2 and HCoV-NL63 RdRp with LibDock scores of 75 and 74, respectively. The LibDock scores of remdesivir to SARS-CoV-2 and HCoV-NL63 were 135 and 151, suggesting that remdesivir may have a higher affinity to HCoV-NL63 compared to SARS-CoV-2 RdRp. In cell culture models infected with HCoV-NL63, both favipiravir and remdesivir significantly inhibited viral replication and production of infectious viruses. Overall, remdesivir compared to favipiravir is more potent in inhibiting HCoV-NL63 in cell culture. Importantly, there is no evidence of resistance development upon long-term exposure to remdesivir. Furthermore, combining favipiravir or remdesivir with the clinically used antiviral cytokine interferon-alpha resulted in synergistic effects. These findings provided a proof-of-concept that anti-SARS-CoV-2 drugs, in particular remdesivir, have the potential to be repurposed for treating HCoV-NL63 infection.

## Introduction

Coronaviruses are enveloped, single-stranded positive sense RNA viruses. There are seven species of coronaviruses known to infect humans. Among them, the highly pathogenic members including MERS-CoV, SARS-CoV and SARS-CoV-2 can cause severe acute respiratory diseases, whereas the seasonal coronaviruses including OC43, HKU1, 229E and NL63 usually cause mild and self-limiting respiratory tract infections^1,2^. The global incidence of upper respiratory diseases is several billion episodes per year, and over 5% are attributed to the infection of these endemic seasonal coronaviruses^1^. Although seasonal coronaviruses are initially thought to be universally low pathogenic, systematic analysis of reported clinical studies have estimated about 25% infected patients can actually develop pneumonia^3^. Importantly, fatality has been reported among healthy adults infected with seasonal coronaviruses^4^.

Human coronavirus NL63 (HCoV-NL63) was first isolated from a 7-month-old child suffering from bronchiolitis and conjunctivitis in The Netherlands^5^. Interestingly, HCoV-NL63 is the only member of seasonal coronaviruses that utilizes angiotensin converting enzyme 2 (ACE2) as its receptor for viral entry^6^, similar to SARS-CoV and SARS-CoV-2. HCoV-NL63 mainly affects young children and immunocompromised patients, resulting in mild upper respiratory symptoms or serious lower respiratory tract complications such as bronchiolitis, croup and pneumonia^7^. Fatal cases have been reported in association with HCoV-NL63 infection^4^. Given that no specific treatment is available, there is an urgent need of developing antiviral therapies against seasonal coronaviruses including HCoV-NL63.

Until the emergence of COVID-19 pandemic caused by SARS-CoV-2, little attention has been paid to seasonal coronaviruses. In response to the pandemic, there are unprecedented efforts in developing antiviral therapies against SARS-CoV-2 infection^2^. We postulate that this represents a unique opportunity for identifying potential therapies against seasonal coronaviruses, for example by repurposing anti-SARS-CoV-2 medications, as these coronaviruses share close genetic relations in several viral genes. Favipiravir is approved for treating influenza virus infection in Japan, and has been shown to inhibit a wide range of viruses including SARS-CoV-2 in experimental models^8–11^. Favipiravir has been authorized in some countries for restricted emergency use of treating COVID-19 patients^12^. Remdesivir is also a broad-spectrum antiviral agent, inhibiting many viruses including MERS-CoV, SARS-CoV and SARS-CoV-2^9,13–15^. Remdesivir has become the first FDA-approved antiviral drug for treating hospitalized COVID-19 patients^16^. Both favipiravir and remdesivir act on RNA-dependent RNA polymerase (RdRp) to inhibit viral replication. In this study, we aim to explore the feasibility of repurposing favipiravir and remdesivir for treating HCoV-NL63 infection. We first comparatively assessed the binding affinities of favipiravir or remdesivir to SARS-CoV-2 and HCoV-NL63 RdRp by molecular docking. Furthermore, we investigated their antiviral activities in cell culture models infected with HCoV-NL63, as well as the combinatory effects with the clinically used antiviral cytokine interferon-alpha (IFN-α).

## Materials and methods

### Protein–ligand docking

Molecular docking of SARS-CoV-2 and HCoV-NL63 RdRp with favipiravir (PubChem CID 492405) or remdesivir (PubChem CID 121304016) was performed with the LibDock program in Discovery Studio Client v20.1.0.19295 (DS, Accelrys Software Inc./BIOVIA, San Diego, CA). LibDock is a flexible docking module^17^, which uses protein site features, referred to as hot spots, consisting of two states (polar and apolar). The ligand poses are placed into the polar and apolar receptor interaction sites. A polar hotspot is preferred by a polar ligand atom (e.g., a hydrogen bond donor or acceptor), and an apolar hotspot is preferred by an apolar atom (e.g., a carbon atom). The protocol allows the user to specify several modes for generating ligand conformations for docking. Conformer Algorithm based on Energy Screening and Recursive buildup (CAESAR) was used for generating the conformations^18^. The smart minimizer was used for in situ ligand minimization. To identify definite interacting residues of the receptor with the bound ligand, a 2D diagram of receptor-ligand interactions was constructed. Scoring was performed using various scoring functions including Jain, Ludi, the potential of mean force (PMF), and piecewise linear potential (PLP1) to evaluate ligand binding in the receptor cavity.

### Molecular dynamics simulation

The docked complexes of SARS-CoV-2/HCoV-NL63 RdRp with favipiravir or remdesivir were used as the initial starting point for the MD (Molecular Dynamics) simulation study for assessing their stability in terms of Root Mean Square Deviation (RMSD) and other parameters. The stability of these protein–ligand complexes was analyzed using the RMSD of the backbone atoms. The dynamics was performed in DS under Chemistry at HARvard Macromolecular Mechanics (CHARMM) 27 force field for protein^19,20^. In the beginning, the complexes were placed into an orthorhombic box and solvated in water for simulation by the Explicit Periodic Boundary solvation model in DS. Later, the heating and equilibration steps were performed at an initial temperature of 50 K for 4 ps and 10 ps, respectively. The temperature was then raised to the target temperature 300 K. The final MD simulation (production step) of the systems was performed for 1000 ps with an NPT (normal pressure and temperature) system at the constant temperature of 300 K and results were saved with a time step of 2 fs and at a collection interval of 20 ps, respectively.

### Reagents and antibodies

Favipiravir (2778-5, BioVision, USA) was dissolved in dimethyl sulfoxide (DMSO, D8418, Sigma, Zwijndrecht, The Netherlands). Remdesivir (HY-104077, MedChem Express, USA) and human IFN-α (H6166, Sigma-Aldrich, The Netherlands) were dissolved in phosphate-buffered saline (PBS, 10572193, Thermo Fisher Scientific). Anti-double-stranded-RNA (dsRNA) antibody (SCIONS J2 monoclonal antibody, 10010200) was purchased from English & Scientific Consulting Kft. Anti-mouse IgG (A32742, H&l Alexa Fluor^®^594, Thermo Fisher Scientific) was used as secondary antibody.

### Viruses and cell lines

Monkey LLC-MK2 cells were cultured in minimal essential medium with Earle’s salt (MEM; Gibco, Grand Island, USA) containing 8% (vol/vol) heat-inactivated fetal calf serum (FCS, Sigma–Aldrich, St. Louis USA), 100× nonessential amino acid (Sciencell, San Diego, California, USA), 200 mM l-Glutamine (Lonza, Verviers, Belgium), 100 IU/mL Penicillin and 100 mg/mL Streptomycin (Gibco, Grand Island, USA). Human colon cancer cell line Caco-2 cells were cultured in Dulbecco’s modified Eagle’s medium (DMEM; Lonza, Verviers, Belgium) containing 10% (vol/vol) heat-inactivated fetal calf serum (FCS, Sigma–Aldrich, St. Louis USA), 100 IU/mL Penicillin and 100 mg/mL Streptomycin (Gibco, Grand Island, USA). Human lung cancer cell line Calu-3 cells were cultured in advanced DMEM/F12 supplemented with 1% (vol/vol) GlutaMAXTM Supplement (Gibco, Grand island, USA), 10 mM HEPES. HCoV-NL63 stock was produced by consecutively inoculating the virus onto LLC-MK2 cells. A humidified incubator was used for cells culturing at 33 °C, with 5% CO_2_. SARS-CoV-2 (isolate BetaCoV/Munich/BavPat1/2020; European Virus Archive Global #026V-03883, GenBank: MT270101) was kindly provided by Dr. Bart Haagmans (Department of Viroscience, Erasmus MC). SARS-CoV-2 stock was produced by consecutively inoculating the virus onto Calu-3 cells. Cell lines were analyzed by genotyping and confirmed to be mycoplasma negative.

### Antiviral drug treatment

LLC-MK2 or Caco-2 cells were first inoculated with HCoV-NL63 at a multiplicity of infection (MOI) of 0.1, and incubated at 33 °C overnight and Calu-3 cells were first inoculated with SARS-CoV-2 at an MOI of 0.02, and incubated at 37 °C for 1 h. The cells were then washed twice with PBS to remove free virus particles and treated with favipiravir or remdesivir for the indicated time period. Cells, total RNA or supernatant were collected for further analysis.

### RNA isolation, cDNA synthesis and qRT-PCR

Total RNA was isolated using Macherey–Nagel NucleoSpin^®^ RNA II kit (Bioke, Leiden, The Netherlands) and quantified using a Nanodrop ND-1000 (Wilmington, DE, USA). cDNA was synthesized by using a cDNA synthesis kit (TaKaRa Bio, Inc., Shiga, Japan). Real-time PCR reactions were performed with SYBR-Green-based real-time PCR (Applied Biosystems^®^, Austin, USA) on a StepOnePlusTM System (Thermo Fisher Scientific LifeSciences). Glyceraldehyde 3-phosphate dehydrogenase (GAPDH) gene was used as housekeeping gene. Relative gene expression of target gene was normalized to GAPDH using the formula 2^−∆∆CT^, ∆∆CT = ∆CTsample − ∆CTcontrol (∆CT = CT [target gene] − CT[GAPDH]). Template control and reverse transcriptase control were included in all qRT-PCR experiments, and all primers are listed in Supplementary Table 1.

### Statistics analysis

All numerical results were reported as mean ± SD. The statistical significance of differences between means was assessed with the Mann–Whitney test (GraphPad Prism 8; GraphPad Software Inc., La Jolla, CA). The threshold for statistical significance was defined as *P* ≤ 0.05.

Note: see additional methods in supplementary file.

## Results

### Modelled structure of HCoV-NL63 RdRp

The homology-based 3D structure of HCoV-NL63 RdRp was modelled in MODELLER 9.24^21^ based on the SARS-CoV-2 RdRp electron microscopic structure with a resolution of 2.93 Å. This produced a total of 20 models for HCoV-NL63 RdRp. Firstly, we selected the model NL63.B99990003 with the least DOPE (discrete optimized protein energy) score for further refinement and study (Table S2). Remarkably, after the GalaxyWEB^22^ refinement process and several stereochemical analyses, we obtained a near native model satisfying all the key parameters at best, and we finally selected the HCoV-NL63 RdRp MODEL 3 (Fig. S1A and Table S3) as the best modelled structure for further docking studies. The structure of MODEL 3 (Fig. S1A) has a Ramachandran value of 97.4% for residues in the most favored region in the plot while with no outlier residues (Fig. S2A), − 2.03 as QMEAN quality estimate score (Fig. S2B) and an enhanced quality factor score of 94.25 for ERRAT analysis (Fig. S2C)^23^. Lastly, we comparatively analyzed the modelled structure HCoV-NL63 RdRp MODEL 3 and the template structure SARS-CoV-2 RdRp from PDB 7C2K. The RMSD between the two structures was 0.386 Å, showing that they share high similarities (Fig. S1B).

### Molecular docking of favipiravir and remdesivir with SARS-CoV-2 and HCoV-NL63 RdRp

**Figure 1:**
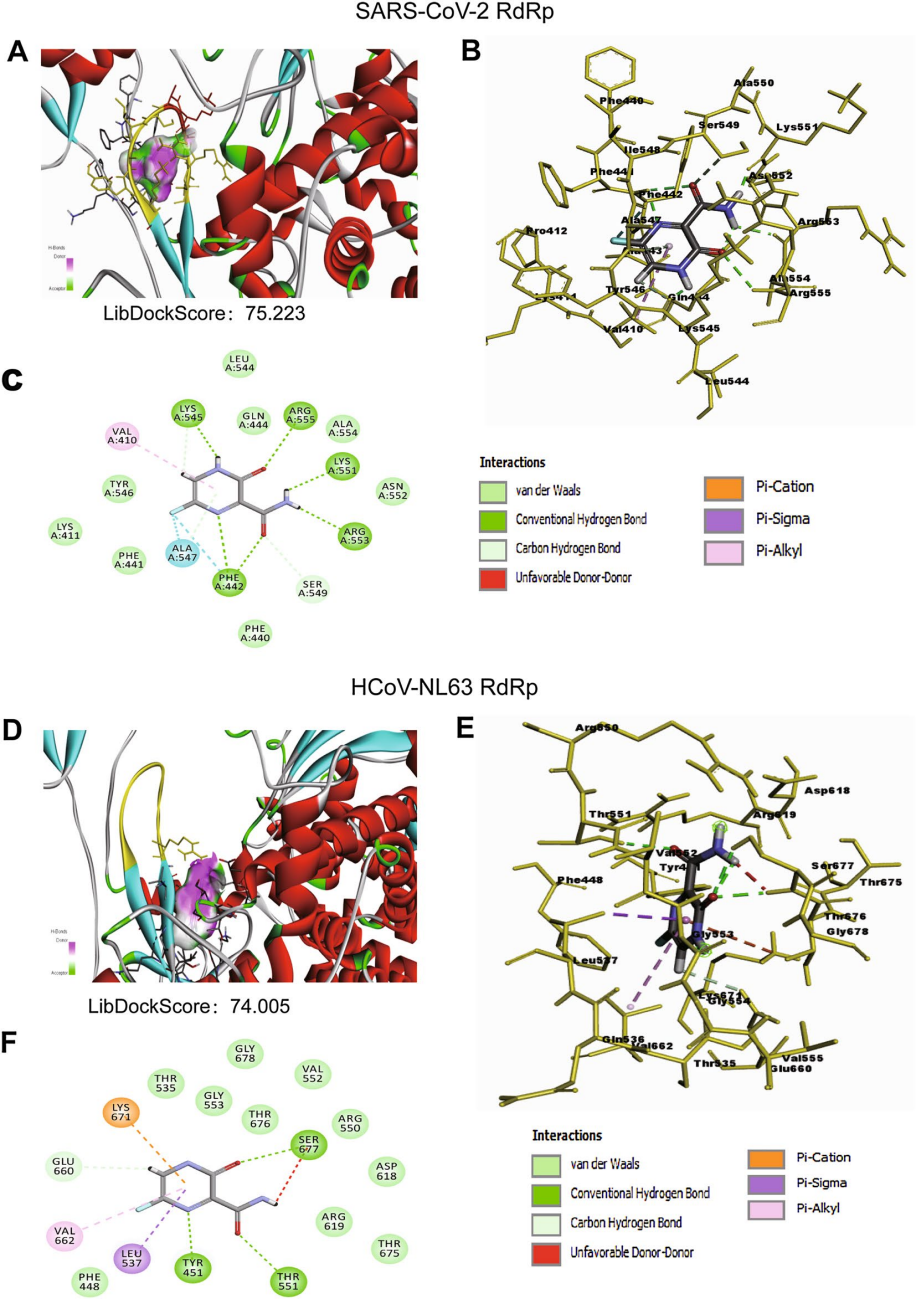
Binding mode of favipiravir to the SARS-CoV-2 and HCoV-NL63 RdRp. (**A**) Favipiravir, bound to the SARS-CoV-2 RdRp (atom color ribbons) binding site, was depicted as surface representation with H-bond donor (purple) and acceptor (green). (**B**) Molecular interactions of favipiravir (atom color sticks) with the SARS-CoV-2 RdRp residues (yellow sticks). (**C**) 2D diagram of interactions between SARS-CoV-2 RdRp and favipiravir. (**D**) Favipiravir, bound to the HCoV-NL63 RdRp (atom color ribbons) binding site, was depicted as surface representation with H-bond donor (purple) and acceptor (green). (**E**) Molecular interactions of favipiravir (atom color sticks) with the HCoV-NL63 RdRp residues (yellow sticks). (**F**) 2D diagram of interactions between HCoV-NL63 RdRp and favipiravir.

**Figure 2:**
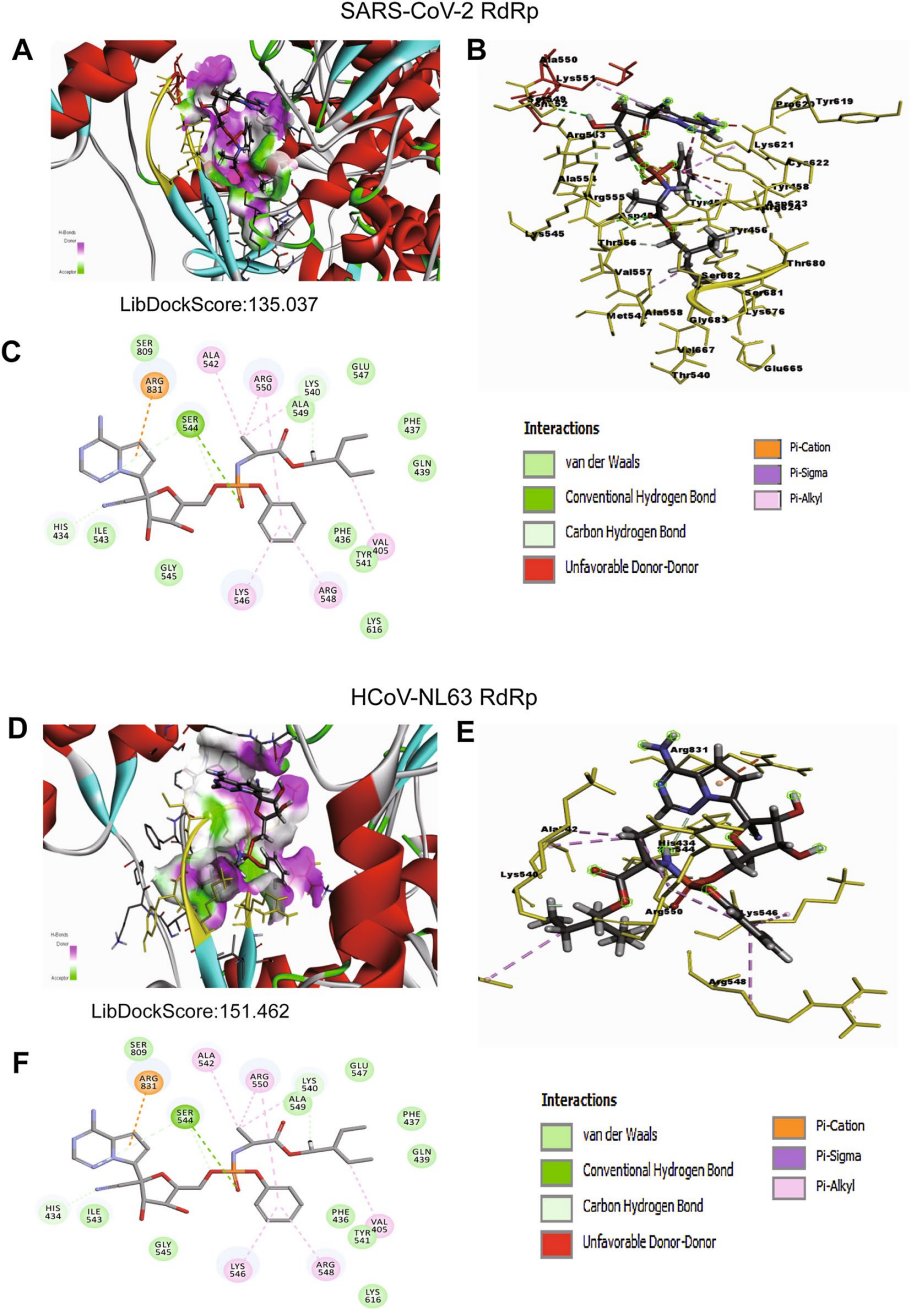
Binding mode of remdesivir to the SARS-CoV-2 and HCoV-NL63 RdRp. (**A**) Remdesivir, bound to the SARS-CoV-2 RdRp (atom color ribbons) binding site, was depicted as surface representation with H-bond donor (purple) and acceptor (green). (**B**) Molecular interactions of remdesivir (atom color sticks) and the SARS-CoV-2 RdRp residues (yellow sticks). (**C**) 2D diagram of interactions between SARS-CoV-2 RdRp and remdesivir. (**D**) Remdesivir, bound to the HCoV-NL63 RdRp (atom color ribbons) binding site, was depicted as surface representation with H-bond donor (purple) and acceptor (green). (**E**) Molecular interactions of remdesivir (atom color sticks) and the HCoV-NL63 RdRp residues (yellow sticks). (**F**) 2D diagram of interactions between HCoV-NL63 RdRp and remdesivir.

Our modelled HCoV-NL63 RdRp structure and the SARS-CoV-2 RdRp structure retrieved from the PDB database (PDB Id 7C2K) were used for docking favipiravir and remdesivir. A grid was defined for SARS-CoV-2 and HCoV-NL63 RdRp around the active regions of 545 to 555 and 540 to 550, respectively, and directional docking with favipiravir and remdesivir was completed in LibDock module of the DS platform. The LibDock scores of SARS-CoV-2 and HCoV-NL63 RdRp with favipiravir are 75.223 and 74.005, forming one and three hydrogen bonds, respectively (Fig. 1A,B,D,E). Compared to favipiravir, remdesivir appears to have a higher affinity in binding to RdRp. The LibDock score of remdesivir with SARS-CoV-2 and HCoV-NL63 RdRp was 135.037 and 151.462, respectively. Remdesivir formed five conventional hydrogen bonds with the residues of both RdRp, along with other electrostatic interactions such as van der Walls (Fig. 2A,B,D,E). The interaction images and their 2D interaction analysis were produced from the Discovery Studio Visualizer tool (Fig. 1C,F, 2C,F).

### Molecular dynamics simulation of the RdRp–ligand complexes

**Figure 3:**
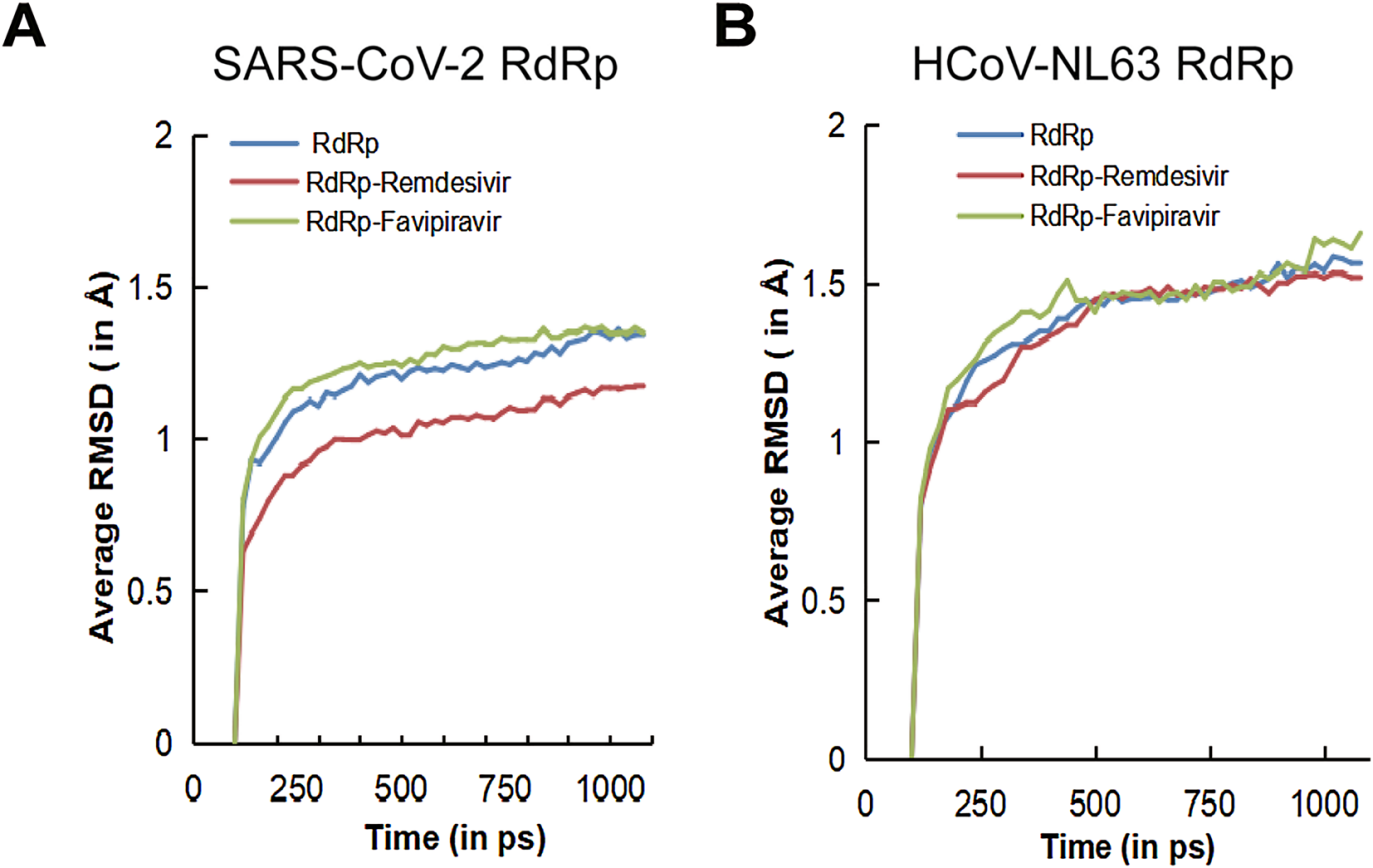
The root-mean-square deviations (RMSDs) of the backbone atoms and its ligands complex relative to their coordinates as a function of simulation time. (**A**) The average RMSDs of SARS-CoV-2 RdRp backbone atoms (Blue line) and its complexes with favipiravir (Green Line) or remdesivir (Red line) were plotted against the total simulation time. (**B**) The average RMSDs of HCoV-NL63 RdRp backbone atoms (Blue line) and its complexes with favipiravir (Green Line) or remdesivir (Red line) were plotted against the total simulation time.

The stability of RdRp–ligand complexes under the dynamic condition was assessed by performing molecular dynamics simulation in the DS platform. The molecular docking complexes of SARS-CoV-2 and HCoV-NL63 RdRp with favipiravir and remdesivir obtained from LibDock module were used to acquire the initial conformations. The simulations were performed for a total time of 1000 ps at a constant temperature of 300 K and their RMSD values for 50 conformations were recorded at a time interval of every 20 ps. The complexes were first heated and equilibrated at an initial temperature of 50 K for 4 ps and 10 ps respectively, before reaching to a final temperature of 300 K for the production (simulation) step. The obtained RMSD values from 50 conformations of SARS-CoV-2 and HCoV-NL63 RdRp structures and their complexes with favipiravir and remdesivir were plotted against the total simulation time for the analysis of complex stability in terms of their RMSD values (Fig. 3A,B). The conformations of SARS-CoV-2 RdRp backbone atoms and its complex with favipiravir and remdesivir have all achieved stability and converged at the end of the simulation (Fig. 3A). The average RMSD of RdRp and its complexes with favipiravir or remdesivir complexes were all below 2 Å with values of 1.18, 1.23 and 1.01 respectively. In HCoV-NL63 RdRp receptor and ligands complex simulation, a slight fluctuation was observed in favipiravir complex at 440 ps and 980 ps of simulation. However, the receptor and complexes attained their stability and converged at the end of the simulation (Fig. 3B). The average RMSD for HCoV-NL63 RdRp and its complexes with remdesivir or favipiravir were also below 2 Å with values of 1.37, 1.40 and 1.34 respectively. Taken together, our simulation indicates that favipiravir and remdesivir can bind to and interact steadily with SARS-CoV-2 and HCoV-NL63 RdRp.

### Anti-HCoV-NL63 effects of favipiravir in different cell culture models

**Figure 4:**
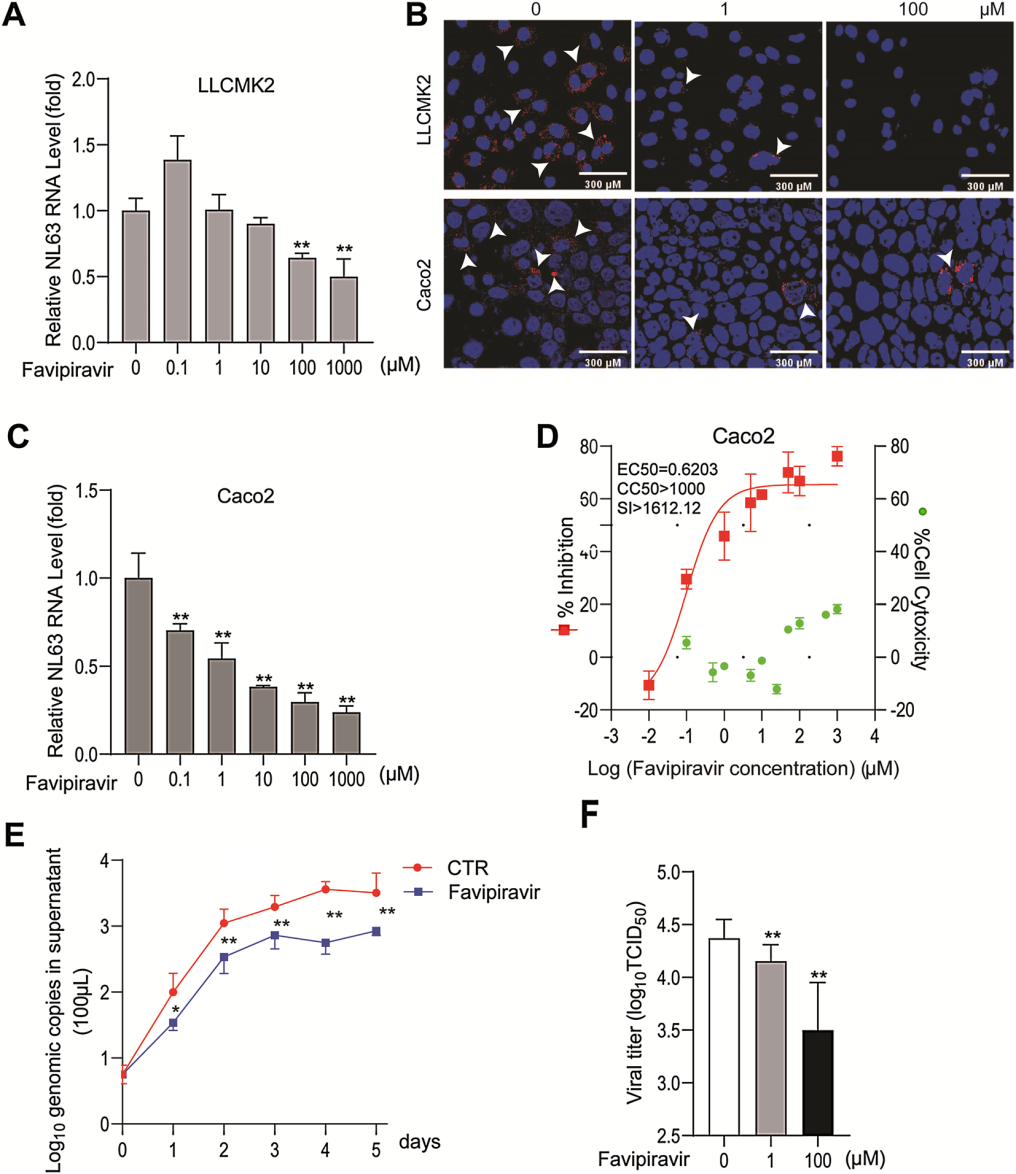
Antiviral effects of favipiravir against HCoV-NL63 in cell culture models. (**A**) Dose-dependent inhibition of HCoV-NL63 replication in LLC-MK2 cell line by favipiravir treatment. Intracellular viral RNA quantified by qRT-PCR was normalized to housekeeping gene GAPDH and presented relative to the control (CTR) (set as 1) (n = 6). (**B**) Immunofluorescence microscopy analysis of dsRNA, the intermediate of replicating HCoV-NL63 genomic RNA, upon treatment of different concentrations of favipiravir in LLC-MK2 and Caco-2 cell lines. Nuclei were visualized by DAPI (blue). (**C**) Dose-dependent inhibition of HCoV-NL63 replication by favipiravir in Caco-2 cell line (n = 6). (**D**) Caco-2 cells were infected with 0.1 MOI HCoV-NL63 and treated with different concentrations of favipiravir for 48 h. 50% effective concentration (EC50) curve was quantified by qRT-PCR, and 50% cytotoxic concentration (CC50) curve was determined by MTT assay. The left and right Y-axis of the graphs represent mean % inhibition of virus yield and cytotoxicity of the drug, respectively (n = 6–8). (**E**) Caco-2 cells were infected with 0.1 MOI HCoV-NL63, and then untreated or treated with 10 μM favipiravir for 5 days. Supernatant was collected every day to quantify secreted viruses by qRT-PCR, calculated as genomic copy numbers (n = 6). Standard curve for calculation of genomic copy numbers is included in Supplementary Fig. S3. (**F**) Caco-2 cells were infected with 0.1 MOI HCoV-NL63, and then untreated or treated with 1 or 100 μM favipiravir for 48 h. Virus titers from different groups was determined by TCID50 assay (n = 6). Data represent as mean ± SD. *P < 0.05; **P < 0.01; ***P < 0.001.

We tested the antiviral effect of favipiravir in LLC-MK2 and Caco-2 cell models infected with HCoV-NL63. Favipiravir dose-dependently inhibited HCoV-NL63 replication determined by qRT-PCR quantification of cellular viral RNA in LLC-MK2 cell model (Fig. 4A), with minimal effects on cell viability (Fig. S3A). This inhibitory effect was further confirmed by immunofluorescent staining of dsRNA, an intermediate of genomic HCoV-NL63 RNA replication (Fig. 4B). In Caco-2 cell model, similar inhibitory effects on viral RNA were observed (Fig. 4B,C) without major effects on cell viability (Fig. S3B). The half maximum effective concentration (EC50) of favipiravir against HCoV-NL63 replication was 0.6203 μM, and the half maximum cytotoxic concentration (CC50) was over 1000 μM, showing a huge selectivity index (SI, CC50/EC50) over 1612 (Fig. 4D).

We further quantified the dynamic effects on secretion of HCoV-NL63 genomic RNA in a consecutive 5-day course by treatment with 10 μM favipiravir in Caco-2 cells. Genomic RNA copy number was calculated as described in Fig. S4. Favipiravir significantly inhibited the release of viral RNA into supernatant (Fig. 4E). For instance, after treating with favipiravir for 4 days, the level of secreted viral RNA resulted in a 84.04% ± 5.23 (mean ± SD, n = 6, *P* < 0.0001) reduction. By harvesting supernatant of Caco-2 cells at 48 h post-treatment of favipiravir, we performed TCID50 assay to determine the titer of secreted infectious viruses. Consistently, the titers of produced HCoV-NL63 with infectivity were significantly decreased by favipiravir (Fig. 4F). For instance, treatment with 100 μM favipiravir reduced 0.87 log10 viral titer and resulted in a 82.06% ± 11.93 (mean ± SD, n = 6, *P* < 0.0001) inhibition.

### Potent inhibition of HCoV-NL63 by remdesivir in cell culture models

**Figure 5:**
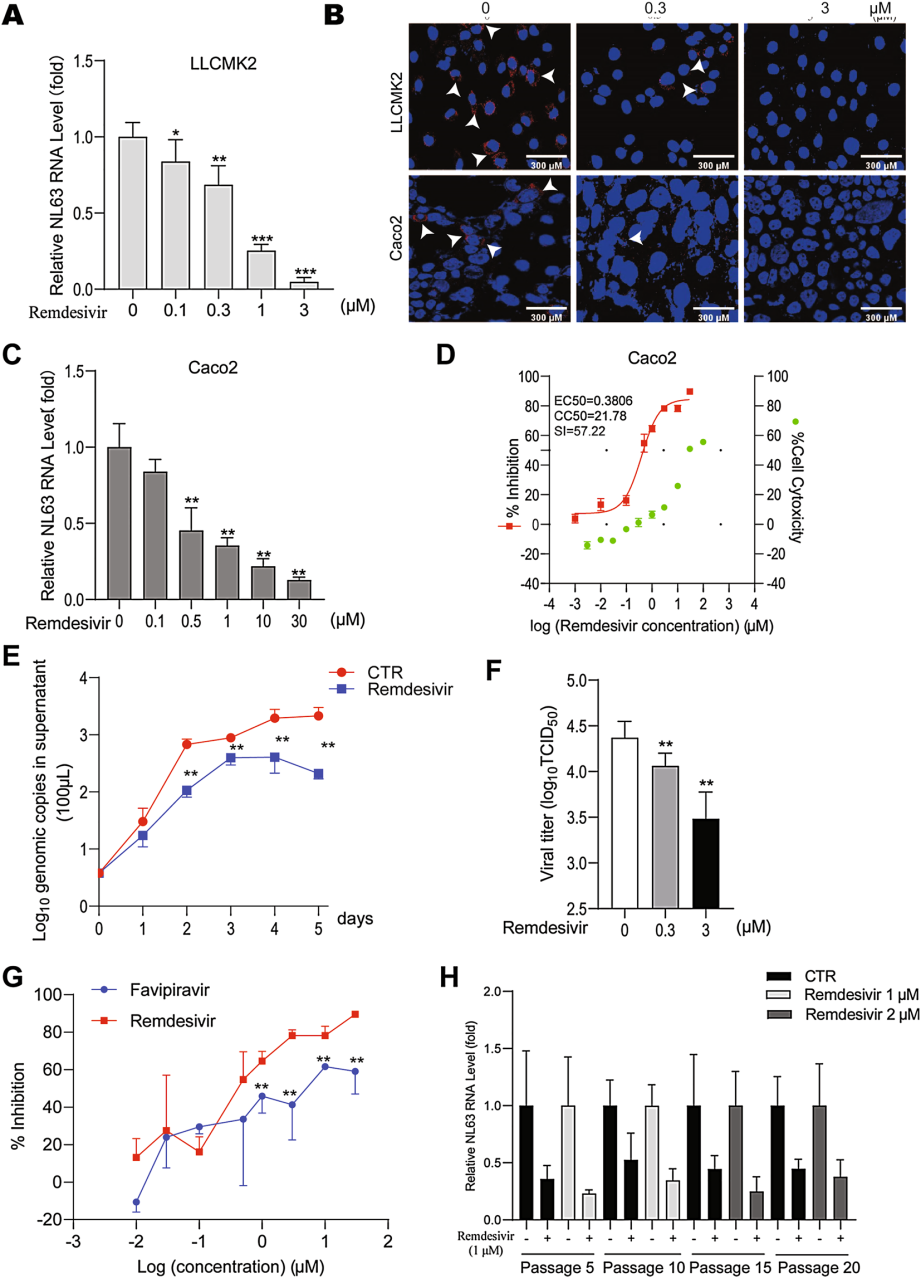
Antiviral effects of remdesivir against HCoV-NL63 in vitro. (**A**) Dose-dependent inhibition of HCoV-NL63 replication by remdesivir in LLC-MK2 cell line. Intracellular viral RNA quantified by qRT-PCR was normalized to housekeeping gene GAPDH and presented relative to the control (CTR) (set as 1) (n = 6–8). (**B**) Immunofluorescence microscopy analysis of dsRNA, the replicating HCoV-NL63 genomic RNA, upon treatment of different concentrations of remdesivir in LLC-MK2 and Caco-2 cell lines. Nuclei were visualized by DAPI (blue). (**C**) Dose-dependent inhibition of HCoV-NL63 replication by remdesivir on in Caco-2 cell line (n = 6). (**D**) Caco-2 cells were infected with 0.1 MOI HCoV-NL63 and treated with different concentrations of remdesivir for 48 h. 50% effective concentration (EC50) curve was quantified by qRT-PCR, and 50% cytotoxic concentration (CC50) curve was determined by MTT assay. The left and right Y-axis of the graphs represent mean % inhibition of virus yield and cytotoxicity of the drug, respectively (n = 6–8). (**E**) Caco-2 cells were infected with 0.1 MOI HCoV-NL63, and then untreated or treated with 1 μM remdesivir for 5 days. Supernatant was collected every day to quantify secreted viruses by qRT-PCR and calculated as genomic copy numbers (n = 6). Standard curve for calculation of genomic copy numbers is included in Supplementary Fig. S3. (**F**) Caco-2 cells were infected with HCoV-NL63 at an MOI of 0.1, then untreated or treated with 0.3 or 3 μM remdesivir for 48 h. Virus titers from different groups was determined by TCID50 assay (n = 6). Data represent as mean ± SD. *P < 0.05; **P < 0.01; ***P < 0.001. (**G**) Comparing the inhibitory potency of favipiravir and remdesivir in Caco-2 cells infected with HCoV-NL63. (**H**) HCoV-NL63 was serially passaged in Caco-2 cells exposed to no remdesivir (as control) or increasing concentrations of remdesivir for 20 passages. 1 μM remdesivir was used in passage 1–10, which was increased to 2 μM at the subsequent passages. The effect of remdesivir (1 μM) on HCoV-NL63 harvested at passage 5, 10, 15 and 20 was quantified using qRT-PCR. Data represent as mean ± SD. *P < 0.05; **P < 0.01; ***P < 0.001.

Remdesivir dose-dependently inhibited HCoV-NL63 replication in LLC-MK2 (Fig. 5A,B) and Caco-2 (Fig. 5B,C) cells. For example, treatment with 30 μM remdesivir for 48 h decreased intracellular virus RNA level by 89.62% ± 1.45 (mean ± SD, n = 6, *P* < 0.0001) in Caco-2 cells. Of note, high dose treatment with remdesivir had moderate effects on cell viability (Fig. S3C and S3D). The EC50 value of remdesivir against HCoV-NL63 replication was 0.3806 μM, the CC50 value was 21.78 μM, and the SI was 57.22 (Fig. 5D). Treatment with 1 μM remdesivir continuously inhibited the secretion of HCoV-NL63 viral RNA over 5 days (Fig. 5E). TCID50 assay showed significant inhibition of viral titers secreted into supernatant by 48 h treatment of remdesivir (Fig. 5F). Treatment with 3 μM remdesivir reduced 0.88 log10 viral titer and resulted in a 85.56% ± 8.25 (mean ± SD, n = 6, *P* < 0.0001) inhibition. Overall, the potency of remdesivir compared with favipiravir is significantly higher in inhibiting HCoV-NL63 replication (Fig. 5G).

Next, we evaluated whether such disparity in antiviral potency of favipiravir and remdesivir also exists in SARS-CoV-2 cell culture model. Indeed, we observed that remdesivir is much more potent with respect to antiviral activity against SARS-CoV-2 (Fig. S5), in accordance with our findings on HCoV-NL63.

Importantly, serially passaging of HCoV-NL63 in the presence of escalating concentrations of remdesivir retained the sensitivity to the treatment (Fig. 5H). Taken together, remdesivir potently inhibits HCoV-NL63 with a high barrier for drug resistance development.

### Synergistic effects of combining favipiravir or remdesivir with IFN-α

**Figure 6:**
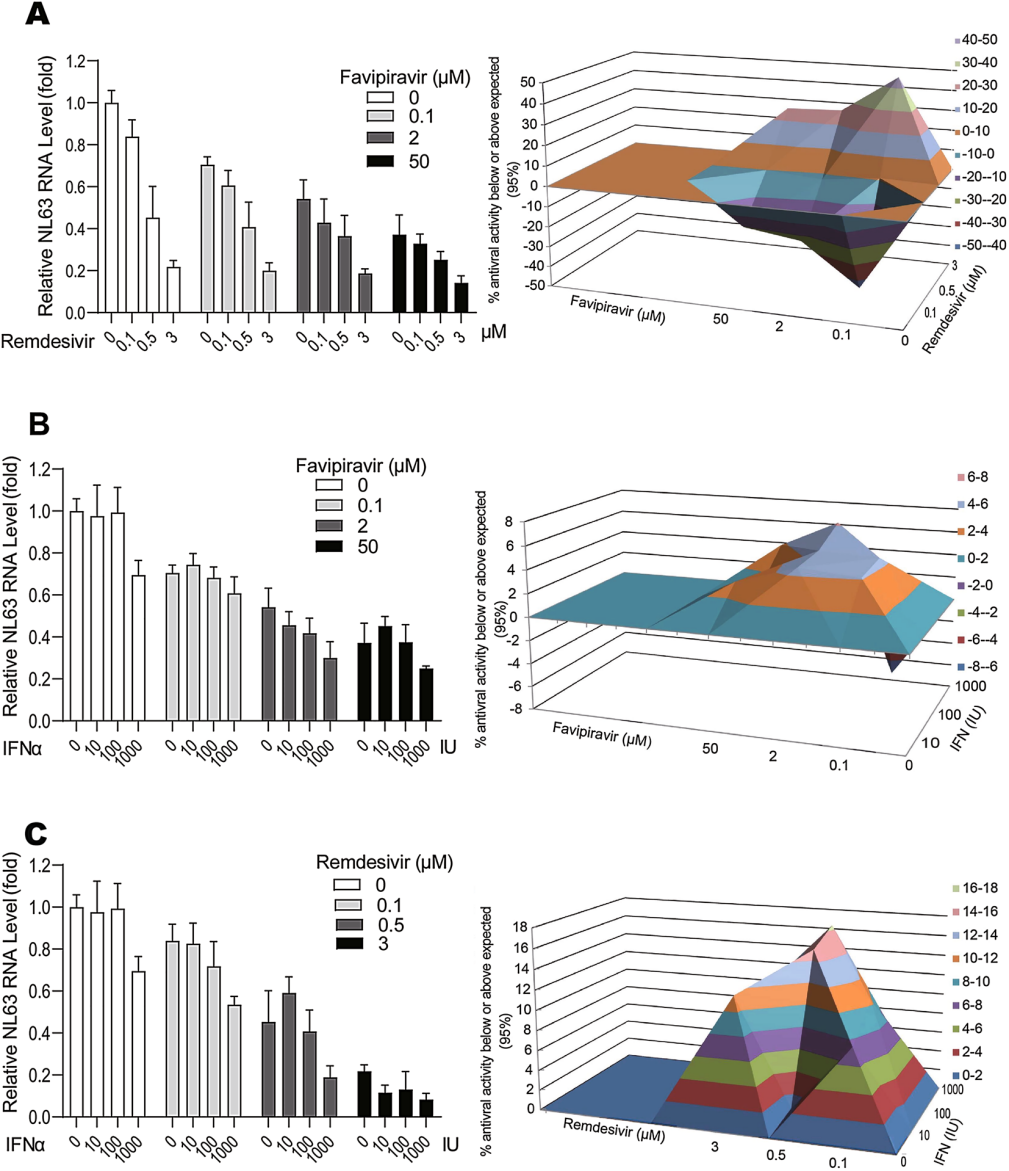
The effects of combining antivirals in Caco-2 cells infected with HCoV-NL63. The antiviral effects of favipiravir in combination with remdesivir (**A**), or IFN-α in combination with favipiravir (**B**) or remdesivir (**C**) respectively. The results were analyzed by the MacSynergyII model. The three-dimensional surface plot represents the differences (within 95% confidence interval) between actual experimental effects and theoretical additive effects of the combination at various concentrations (n = 4).

Combination treatment is often used to enhance antiviral efficacy and to avoid drug resistance development in clinical applications. We evaluated the combined antiviral effects of favipiravir or remdesivir with IFN-α. A moderate antagonistic effect (− 53.96 μM^2^ %) was observed with the combination of favipiravir and remdesivir, implying that they employ a similar antiviral mechanism that both agents bind to HCoV-NL63 RdRp (Fig. 6A). IFN-α, as a widely used antiviral drug, has a distinct mechanism-of-action by activating host antiviral immune response^24^. Since IFN-α has been clinically explored for treating COVID-19 patients^25^, we tested the combinatory effects with favipiravir or remdesivir. Indeed, both favipiravir and remdesivir exhibited moderate synergistic effects with IFN-α (24.52 μM^2^ %, 28.42 μM^2^ %) (Fig. 6B,C). The tested concentrations of the antivirals had little cytotoxicity on host cells (Fig. S6).

## Discussion

Computational simulation has become a powerful tool for mapping the interactions between ligands and target proteins, and therefore is widely used for drug development. Combining molecular docking and molecular dynamics simulation, a wide range of compounds have been profiled for potential interactions with viral proteins of SARS-CoV-2, in particular the RdRp, in order to rapidly identify antiviral drugs for treating COVID-19 patients^26,27^. In this study, we used similar approaches for identifying antiviral agents against HCoV-NL63. Because no experimentally solved crystal structure of HCoV-NL63 RdRp is available, we first modelled its 3D structure based on the experimentally solved structure of SARS-CoV-2 RdRp. This resulted in the identification of a template with an identity of 59.1% covering 99% of HCoV-NL63 RdRp query sequence as template for homology modeling. Through initial modeling and subsequent refinement, we selected the best-scored model of HCoV-NL63 RdRp structure.

The high similarity between the structures of SARS-CoV-2 and HCoV-NL63 RdRp shown by superimposed chimera tool^28^ comparison analysis supported our rationale of repurposing RdRp-targeted anti-SARS-CoV-2 drugs for treating HCoV-NL63 infection. We thus performed molecular docking of the clinically used anti-SARS-CoV-2 drugs favipiravir and remdesivir with the SARS-CoV-2 and HCoV-NL63 RdRp. Based on the formed conformations, we can clearly visualize the binding of the two drugs to the RdRp of the two coronaviruses. LibDock score is a hallmark of binding affinity: the greater the LibDock score, the better the binding affinity^29^. Our simulation indicated a similar affinity of favipiravir in binding to the SARS-CoV-2 and HCoV-NL63 RdRp with LibDock scores of 75 and 74, respectively. The affinity of remdesivir in binding to RdRp appears much stronger in general, compared to that of favipiravir. Interestingly, the LibDock score of remdesivir with HCoV-NL63 RdRp (151) is even higher than that of SARS-CoV-2 RdRp (135). Molecular dynamics simulation further confirmed the stability of favipiravir and remdesivir binding to the SARS-CoV-2 and HCoV-NL63 RdRp.

It is of foremost importance to validate in silico identified drug candidates in experimental models before clinical testing. Unfortunately, during the unprecedented COVID-19 crisis, some in silico molecular docking identified candidates were directly tested in COVID-19 patients, prior to concrete evaluation in experimental models. Inadequate preclinical studies of drug candidates often impose a high risk of failure in clinical trials with substantial safety concerns^2^. In this study, we further validated our in silico results in Caco-2 and LLC-MK2 cell culture models infected with HCoV-NL63. These two cell lines have been widely used for modeling coronavirus infections^30^. We comprehensively demonstrated that both favipiravir and remdesivir significantly inhibit HCoV-NL63 replication and production of infectious virus particles. However, the antiviral effect of remesivir is much potent than favipiravir, which is consistent with our molecular docking results that remdesivir has a better binding affinity to HCoV-NL63 RdRp compared to favipiravir. This is in line with previous observations in cell culture models of SARS-CoV-2 that the EC50 values of favipiravir and remdesivir were 61.88 µM and 0.77 µM, respectively^14^. In a previous study testing remdesivir on SARS-CoV and MERS-CoV, HCoV-NL63 was included as a control model showing inhibitory effect, but it was not studied in detail^31^. In our HCoV-NL63 model, we demonstrated that the EC50 of remdesivir was 0.38 µM, whereas a previous study reported the antiviral activity of remdesivir against the other two seasonal coronaviruses including HCoV-OC43 and HCoV-229E as 0.15 µM and 0.024 µM, respectively^32^. These studies collectively suggest a broad-spectrum antiviral activity of remdesivir against different coronaviruses.

In SARS-CoV-2-infected hamsters, only a high dose of favipiravir treatment which may not be clinically relevant has been shown to exert antiviral activity^33^. In COVID-19 patients, favipiravir treatment failed to improve the recovery rate^34,35^. We thus question the usefulness of exploring favipiravir monotherapy for treating seasonal coronavirus infections. In rhesus macaques infected with SARS-CoV-2, remdesivir treatment reduced both pulmonary infiltrates on radiographs and virus titers in bronchoalveolar lavages 12 h after the first treatment, although virus shedding from the upper respiratory tract was not reduced^36^. This suggests that the anti-SARS-CoV-2 effect of remdesivir may be more prominent at the early stage of infection. The final report of a large double-blind, randomized, placebo-controlled trial has demonstrated that remdesivir treatment significantly shortens the median recovery time from 15 to 10 days in hospitalized COVID-19 patients. The death rate by 29 days was 11.4% with remdesivir treatment compared to 15.2% with placebo, but this numerical reduction did not reach conventional statistical significance^16^. Another clinical trial in patients with severe COVID-19 did not show a significant difference between a 5-day course and a 10-day course of remdesivir treatment. However, due to no placebo control, the magnitude of benefit cannot be determined^37^. These results indicate that remdesivir treatment could be beneficial for severe COVID-19 patients, but the efficacy remains suboptimal.

Seasonal coronaviruses including HCoV-NL63 is imposing a clinical burden in special populations with association of fatalities in some cases^4^. We believe that repurposing anti-SARS-CoV-2 drugs is a viable option for developing therapeutics for patients with infections of seasonal coronaviruses. Based on results from the current and previous studies, remdesivir has the potential to be further explored in this respect. However, remdesivir monotherapy is likely not sufficient to completely cure the patients, and combination with other antivirals may be necessary to enhance the efficacy. In this study, we found that the combination of favipiravir and remdesivir had little synergistic antiviral effect. This may be explained by the fact that both drugs target the viral RdRp. IFN-α, a broad-spectrum antiviral cytokine that activates host defense mechanisms, has been used to treat viral infections in the clinic for decades^24^. Clinical trials are currently investigating the combination of IFN-α with different antiviral drugs for treating COVID-19 patients^38^. In our cell culture models of HCoV-NL63, we found that combining favipiravir or remdesivir with IFN-α resulted in synergistic antiviral effects. Thus, we postulate that drug combination strategies deserve to be further explored for treating coronavirus infections, based on an in-depth understanding of the different mechanisms-of-actions of the drug candidates. This shall enhance the antiviral efficacy but also mitigate the risk of resistant mutation emergence.

In summary, we have successfully modelled the 3D structure of HCoV-NL63 RdRp. Through molecular docking, we found that both favipiravir and remdesivir can bind to HCoV-NL63 RdRp, but remdesivir has a higher binding affinity. In cell culture models, favipiravir and remdesivir significantly inhibited HCoV-NL63 infection, whereas remdesivir exerted higher antiviral potency. Importantly, the combination of favipiravir or remdesivir with IFN-α resulted in synergistic antiviral effects. These findings provided a new scenario of developing antiviral therapies against seasonal coronavirus infection through repurposing anti-SARS-CoV-2 medications. We expect that remdesivir compared to favipiravir is likely to have a better potential for exploration in this respect, but further in vivo evaluation is warranted to confirm our findings.

## Supplementary Information

Supplementary Information.
