# Supplementary material for: Comparative assessment of favipiravir and remdesivir against human coronavirus NL63 in molecular docking and cell culture models
Source: Sci Rep. 2021 Dec 6;11:23465. doi: 10.1038/s41598-021-02972-y (PMC8648821; doi:10.1038/s41598-021-02972-y)
Supplement: Supplementary file 1 — Supplementary Information. [file 41598_2021_2972_MOESM1_ESM.pdf]

Supplementary information to

Comparative assessment of favipiravir and remdesivir against human coronavirus NL63 in molecular docking and cell culture models

Yining Wang, Pengfei Li, Sajjan Rajpoot, Uzma Saqib, Peifa Yu, Yunlong Li, Yang Li, Zhongren Ma, Mirza S. Baig, Qiuwei Pan

Supplementary Materials and methods

3D protein structure modeling of HCoV-NL63 RdRp

A homology-based approach was applied to model the 3D protein structure of HCoV-NL63 RdRp. We used the MODELLER 9.24 for modeling and loop refinement of the structure. The full-length sequence of HCoV-NL63 RdRp was obtained from the NCBI protein database (Accession AIW52828.1) and protein-protein BLAST tool was used for finding the most similar protein structure in the PDB database.

The structure of SARS-CoV-2 RdRp (PDB Id 7C2K) with highest similarity was retrieved from the PDB database, and its Chain A was used as a template for modeling. The MODELLER 9.24 program was run using the query and template sequence alignment file in PIR format. The validation of the modelled structure was performed using PROCHECK and ERRAT programs. The outlier’s residues in the

Ramachandran plot were corrected through loop refinement in MODELLER 9.24. DOPE (discrete optimized protein energy) score was used to select the best model for further *in silico* stereochemical quality assessment and docking studies. Finally, GalaxyWEB was used for overall structure refinement of the modelled protein. The final model of protein structure was re-analyzed by Swiss-model structure assessment (generating the Ramachandran plot and QMEAN score), PROCHECK, and ERRAT. Chimera tool was used to perform the superimposition for comparative analysis of target and template structure to calculate the root mean square deviation (RMSD) and to energetically minimize the structure for docking studies. BIOVIA Discovery studio visualizer was used for image preparation of model structures.

Quantification of HCoV-NL63 genome copy numbers

An amplicon of the HCoV-NL63 (primers are listed in Supplementary table 1) was cloned into the pCR2.1-TOPO vector (Invitrogen, San Diego, CA) to generate a template for quantifying HCoV-NL63 genome copy numbers. The plasmid was extracted by Quick Plasmid Miniprep Kit (Invitrogen, Lohne, Germany). A series of dilutions (from 10^{-1} to 10^{-8}) were prepared and then were amplified and quantified by qRT-PCR to generate a standard curve. This standard curve was generated by plotting the log copy number versus the cycle threshold (CT) value (Supplementary figure S1). Copy numbers were calculated by using the following equation: Copy number (molecules/μl) = [concentration (ng/μL) × 6.022×10^{23} (molecules/mol)]/

[length of amplicon× 640 (g=/mol) × 10^9 (ng/g).

Confocal fluorescence microscopy

LLC-MK2 or Caco-2 cells cultured in an 8-well chamber (cat. no. 80826; ibidi GmbH) were inoculated with HCoV-NL63 at 0.1 MOI, and incubated at 33 °C overnight. The culture medium was then replaced by medium containing different concentrations of favipiravir or remdesivir, and the cells were cultured for another 48 hours. The cells were fixed with 4% paraformaldehyde in PBS for 10 min, permeablized with 0.2% (vol/vol) Triton X-100 for 10 min, blocked with milk-tween-glycine medium (0.05% tween, 0.5% skim milk and 0.15% glycine) for 1 hour, and reacted with anti-dsRNA antibody (1:500) diluted in blocking solution at 4 °C overnight. The cells were then incubated with 1:1000 dilutions of the anti-mouse IgG secondary antibodies for 1 hour. Nuclei were stained with DAPI (4, 6-diamidino-2-phenylindole; Invitrogen). Images were detected using Leica SP5 cell imaging system.

TCID50 assay

Viruses in the cultured cells the supernatant were harvested through repeated freezing and thawing for three times. HCoV-NL63 titer was quantified by using a 50% tissue culture infectious dose (TCID50) assay. Briefly, ten-fold dilutions of HCoV-NL63 were inoculated onto LLC-MK2 cells grown in a 96-well tissue culture plate at 1,000 cells/well. The plate was incubated at 33 °C for 5-7 days, and each well was

examined under a light microscope for cytopathic effect (CPE). The TCID50 value was calculated by using the Reed-Muench method.

MTT assay

LLC-MK2 or Caco-2 cells were seeded into 96-well tissue culture plates (1×10^4cells/well), and then treated with the indicated compounds for 48 hours. Cells were incubated with 10 μL 5 mg/mL 3-(4,5-dimethyl-2-thiazolyl) -2,5-diphenyl-2H-tetrazolium bromide (MTT) for 3 hours, then replaced with 100 μL DMSO medium and incubated at 37°C for 30 minutes. The absorbance at 490 nm was recorded using a microplate absorbance reader (Bio-Rad, CA, USA).

Serial passaging of HCoV-NL63 with remdesivir treatment

HCoV-NL63 were passaged in Caco-2 cells in the absence of drug (vehicle control) or in the presence of gradually increasing concentrations of the drug (1 μM of remdesivir for passage 1–10 and 2 μM of remdesivir for passage 11–20). In brief, Caco-2 cells in 6-well plate were inoculated with virus (MOI = 0.5) at 33 °C for overnight, followed by adding remdesivir or without drug (as control). After 48 hours, both cells and supernatant were collected to harvested the virus through freezing and thawing for once, and centrifuged. The supernatant containing passaged viruses was stored at −80 °C until used for the next passage. Viruses were serially passaged by using 1 aliquot of viral stock from the preceding passage to infect fresh

Caco-2 cells. The effect of each passage of virus (same titer) was quantified by qRT-PCR.

Supplementary Table 1. Primers used in the study

Gene name	F-sequence (5' to 3')	R-sequence (5' to 3')
HCoV-NL63	CTTCTGGTGACGCTAGTACAGCTTAT	AGACGTCGTTGTAGATCCCTAACAT
Human GAPDH	GTCTCCTCTGACTTCAACAGCG	ACCACCCTGTTGCTGTAGTAGCCA A
NL63	ACGCAATGCCACTGTTGTTA	GACAACACCGTCATCAGAGA

Supplementary Table 2.

S.No.	NL63 Models (by MODELLERS 9.24)	Dope Score (Sorted as Lowest to Highest)
**1**	**NL63.B99990003.pdb**	**-112701.8906**
2	NL63.B99990001.pdb	-112574.4297
3	NL63.B99990002.pdb	-112572.9141
4	NL63.B99990014.pdb	-112557.8438
5	NL63.B99990019.pdb	-112481.5
6	NL63.B99990018.pdb	-112452.8672
7	NL63.B99990012.pdb	-112417.6016
8	NL63.B99990016.pdb	-112373.3359
9	NL63.B99990011.pdb	-112370.625
10	NL63.B99990006.pdb	-112350.8281
11	NL63.B99990020.pdb	-112286.75

12	NL63.B99990013.pdb	-112247.5859
13	NL63.B99990004.pdb	-112196.8984
14	NL63.B99990015.pdb	-112190.4141
15	NL63.B99990008.pdb	-112073.9688
16	NL63.B99990017.pdb	-112056.4453
17	NL63.B99990010.pdb	-111899.5391
18	NL63.B99990007.pdb	-111876.7969
19	NL63.B99990009.pdb	-111805.25
20	NL63.B99990005.pdb	-111565.0313

Supplementary Table 2. DOPE (discrete optimized protein energy) score of HCoV-NL63 RdRp structure models produced by homology-based modeling by MODELLER 9.24. The DOPE score for 20 HCoV-NL63 RdRp models presented in the table are sorted in order from the lowest to highest. The Model NL63.B99990003 with least DOPE score was selected for further refinement and study.

Supplementary Table 3.

Model	GDT-HA	RMSD	MolProbity	Clash score	Poor rotamers	Ramachand-ran favored(%age)	ERRAT Score
Initial	1.0000	0.000	2.950	71.8	2.7	95.7	72.3872
MODE	0.9822	0.307	1.759	13.2	1.1	97.5	92.21

L1							08
MODEL2	0.9833	0.304	1.704	12.1	1.1	97.6	93.5927
**MODEL3**	**0.9830**	**0.310**	**1.764**	**12.8**	**1.1**	**97.4**	**94.2503**
MODEL4	0.9800	0.313	1.738	13.5	0.7	97.5	91.5235
MODEL5	0.9822	0.305	1.744	12.6	0.6	97.3	92.8328

Supplementary Table 3. List of total five HCoV-NL63 RdRp refined model structure obtained from GalaxyRefine tool of Galaxy WEB Server. The ERRAT score was further calculated for each refined model and represented here in last column of the table. The HCoV-NL63 RdRp model 3 with best average score and quality for stereochemical analysis parameter was selected as final HCoV-NL63 RdRp model structure for docking studies.

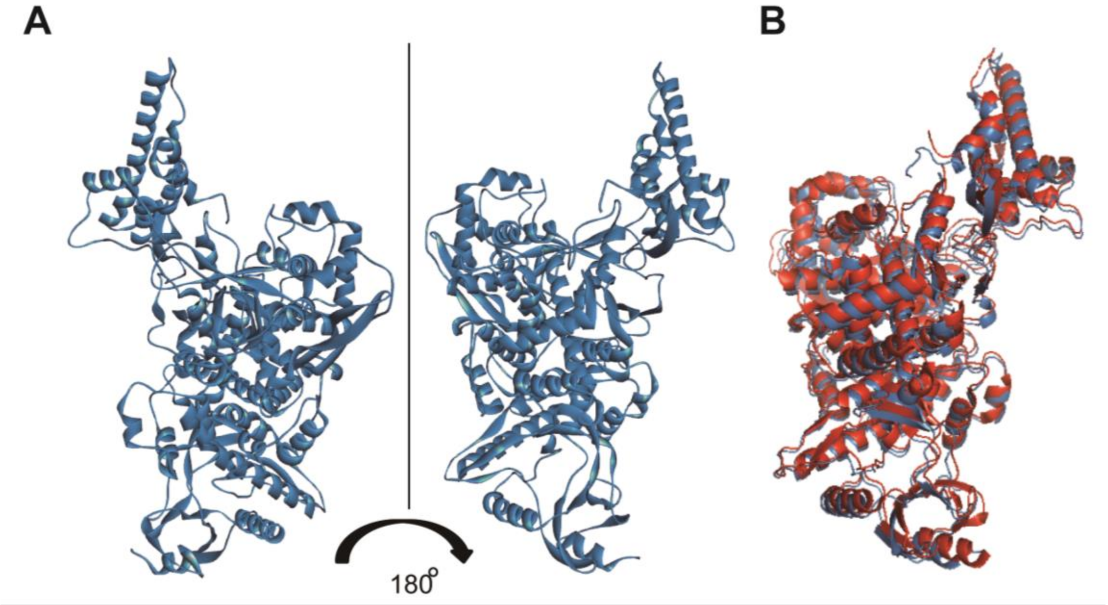

Supplementary Fig. S1. Modeling the 3D structure of HCoV-NL63 RdRp. (A) Modelled structure of HCoV-NL63 RdRp (cyan). (B) Superposition of the modelled structure of HCoV-NL63 RdRp (cyan) with the experimentally solved structure of SARS-CoV-2 RdRp (red).

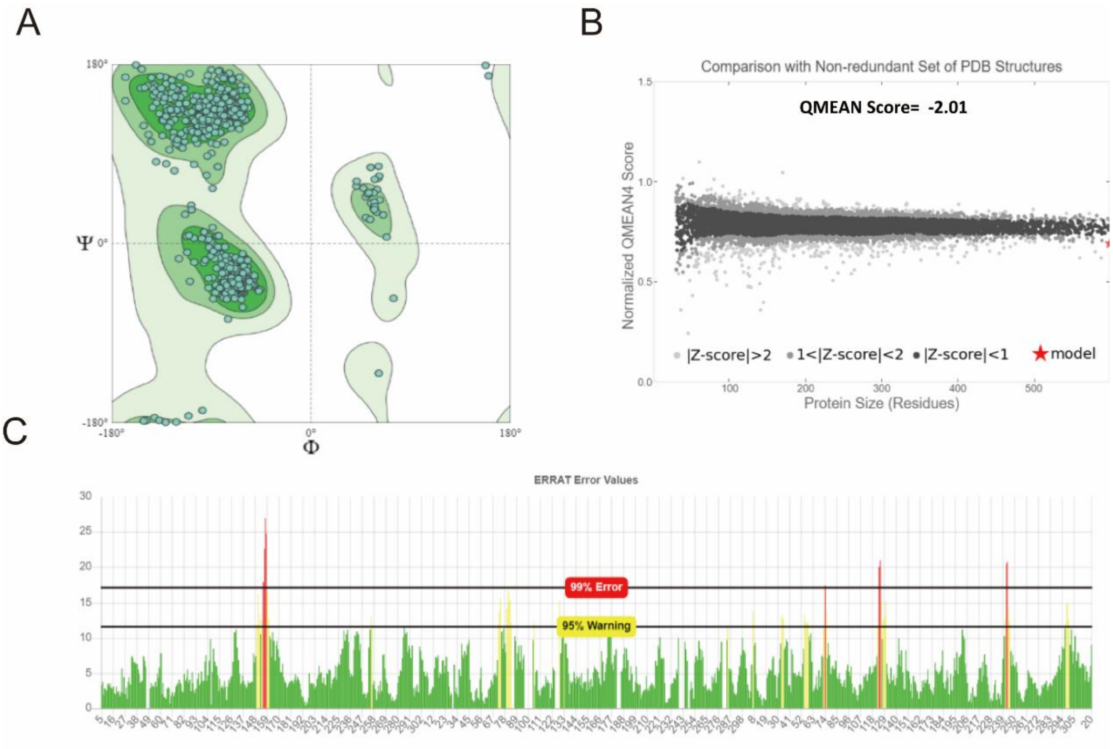

Supplementary Fig. S2. Stereochemical analysis and structural validation of HCoV-NL63 RdRp model MODEL 3. (A) Ramachandran plot produced 97.4% score for the model with no outlier residues in unfavoured region. (B) QMEAN (Qualitative Model Energy ANalysis) quality factor normalized score with total protein size and its Z-score value. (C) ERRAT error value for each residue of the HCoV-NL63 RdRp MODEL 3 which results in overall value of 94.2503 respectively.

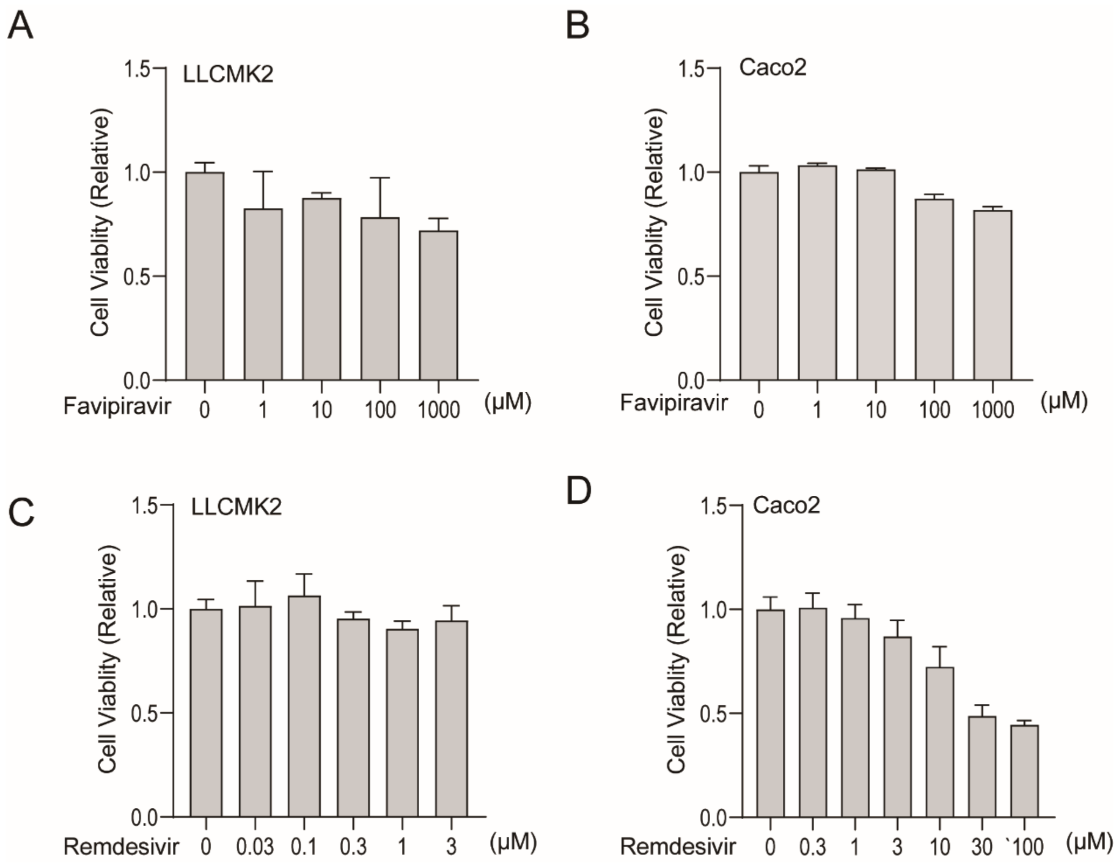

Supplementary Fig. S3. The cytotoxicity of favipiravir or remdesivir on LLC-MK2 and Caco-2 cell lines. (A) LLC-MK2 cells treated with different concentrations of favipiravir for 48 h. Cytotoxicity was determined by MTT assay (n = 6-8). (B) Caco-2 cells were treated with different concentrations of favipiravir for 48 h. Cytotoxicity was determined by MTT assay (n = 6-8). (C) LLC-MK2 cells were treated with different concentrations of remdesivir for 48 h. Cytotoxicity was determined by MTT assay (n = 6-8). (D) Caco-2 cells were treated with different concentrations of remdesivir for 48 h. Cytotoxicity was determined by MTT assay (n = 6-8).

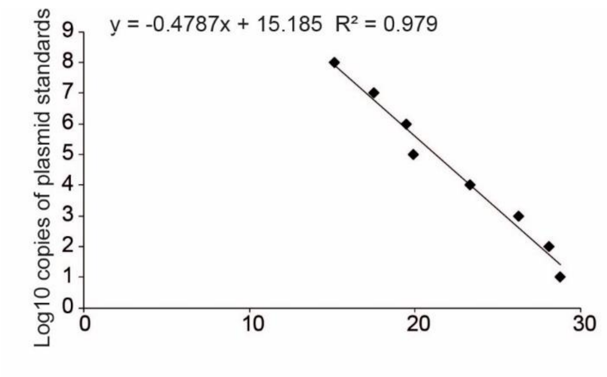

Supplementary Fig. S4. Standard curve for quantifying HCoV-NL63 genome copy numbers. An amplicon of the HCoV-NL63 was cloned into the pCR2.1-TOPO vector. The plasmid was extracted, followed by a series of dilutions from 10^{-1} to 10^{-8} and then were amplified and quantified by qRT-PCR. Standard curve was generated by plotting the cycle threshold (CT) value regarding the log copy numbers

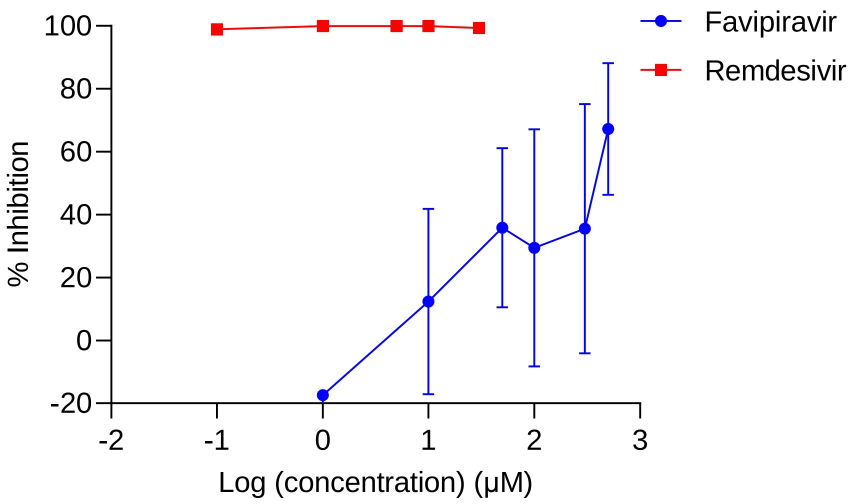

Supplementary Fig. S5. Comparing the inhibitory potency of favipiravir and remdesivir in Calu-3 cells infected with SARS-CoV-2. Calu-3 cells were infected with SARS-CoV-2 at an MOI of 0.02 in the treatment of different concentrations of favipiravir or remdesivir for 48 hours. The viral yield in the cell supernatant was then quantified by qRT-PCR.

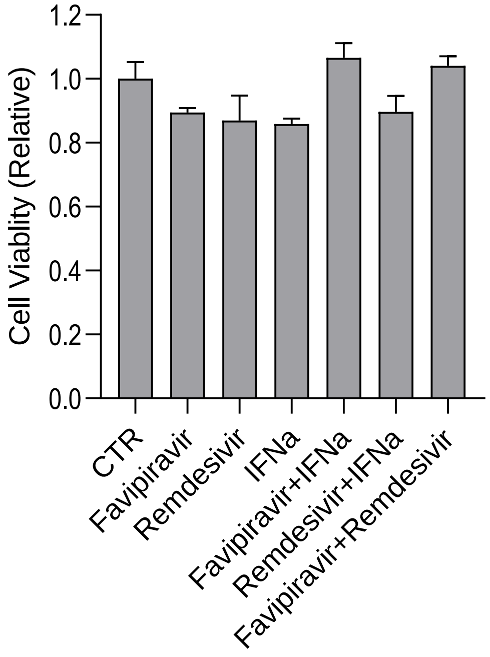

Supplementary Fig. S6. Cytotoxicity of favipiravir, remdesivir, IFN-α or their combinations. Caco-2 cells were treated with different concentrations of favipiravir (50 μM), remdesivir (3 μM), IFN-α (1000 IU) or their combinations respectively for 48 h. Cytotoxicity was determined by MTT assay (n = 8).
